# The Indole-3-Acetamide-Induced *Arabidopsis* Transcription Factor MYB74 Decreases Plant Growth and Contributes to the Control of Osmotic Stress Responses

**DOI:** 10.3389/fpls.2022.928386

**Published:** 2022-06-22

**Authors:** Paloma Ortiz-García, Marta-Marina Pérez-Alonso, Adrián González Ortega-Villaizán, Beatriz Sánchez-Parra, Jutta Ludwig-Müller, Mark D. Wilkinson, Stephan Pollmann

**Affiliations:** ^1^Centro de Biotecnología y Genómica de Plantas,Universidad Politécnica de Madrid (UPM)–Instituto Nacional de Investigación y Tecnología Agraria y Alimentación (INIA /CSIC), Madrid, Spain; ^2^Umeå Plant Science Center, Umeå University, Umeå, Sweden; ^3^Institute of Biology, University of Graz, Graz, Austria; ^4^Institute of Botany, Technische Universität Dresden, Dresden, Germany; ^5^Departamento de Biotecnología-Biología Vegetal, Escuela Técnica Superior de Ingeniería Agronómica, Alimentaria y de Biosistemas, Universidad Politécnica de Madrid (UPM), Madrid, Spain

**Keywords:** auxin, plant hormone crosstalk, growth repression, plant stress response, *Arabidopsis thaliana*

## Abstract

The accumulation of the auxin precursor indole-3-acetamide (IAM) in the *ami1* mutant has recently been reported to reduce plant growth and to trigger abiotic stress responses in *Arabidopsis thaliana*. The observed response includes the induction of abscisic acid (ABA) biosynthesis through the promotion of *NCED3* expression. The mechanism by which plant growth is limited, however, remained largely unclear. Here, we investigated the transcriptional responses evoked by the exogenous application of IAM using comprehensive RNA-sequencing (RNA-seq) and reverse genetics approaches. The RNA-seq results highlighted the induction of a small number of genes, including the R2R3 MYB transcription factor genes *MYB74* and *MYB102*. The two MYB factors are known to respond to various stress cues and to ABA. Consistent with a role as negative plant growth regulator, conditional *MYB74* overexpressor lines showed a considerable growth reduction. RNA-seq analysis of *MYB74* mutants indicated an association of MYB74 with responses to osmotic stress, water deprivation, and seed development, which further linked MYB74 with the observed *ami1* osmotic stress and seed phenotype. Collectively, our findings point toward a role for MYB74 in plant growth control and in responses to abiotic stress stimuli.

## Introduction

Plants are continually challenged by an ever-changing environment. Many environmental alterations are unfavorable and affect the development of plants negatively. In fact, plants must endure a wide variety of abiotic stress conditions, including drought, salinity, and adverse temperatures. These stressors greatly limit plant growth and productivity, forcing plants to adjust their developmental programs accordingly to cope with environmental variations and ensure the survival of their progeny. Over recent years, our understanding of the multifaceted and comprehensive molecular processes that contribute to orchestrating adequate abiotic stress responses in plants, including stress perception, signal transduction, and transcriptional reprogramming, has made substantial progress but is still far from being complete (Zhang et al., 2022).

Phytohormones are key drivers of plant stress responses. They are well-known small signaling molecules that show quick responses to environmental changes and act at submicromolar concentrations (Davies, 2010). The plant hormones abscisic acid (ABA), salicylic acid (SA), and jasmonic acid (JA) have been recognized as classical stress hormones, as a wealth of studies reported their key roles in plant stress responses. However, there is also mounting evidence that the crosstalk of these stress hormones with the remaining plant hormones, including brassinosteroids, ethylene, and auxin, is an important factor that is reported to determine the adequate level of response (Verma et al., 2016).

Currently, not much is known about the molecular basis of ABA–auxin crosstalk, although several studies point to the cooperation of these two plant hormones in a number of physiological processes (Emenecker and Strader, 2020). For example, both plant hormones inhibit primary root growth in light-grown Arabidopsis seedlings (Thole et al., 2014). Furthermore, a recent study reported that both the endogenous accumulation of the auxin precursor indole-3-acetamide (IAM) and the exogenous application of IAM can trigger ABA biosynthesis through the transcriptional activation of *NCED3* (Pérez-Alonso et al., 2021a). 9-*cis*-Epoxycarotenoid dioxygenases (NCEDs) are key rate-limiting enzymes in the ABA biosynthesis pathway (Huang et al., 2018b). Pérez-Alonso and colleagues (2021) reported that the abiotic stress-mediated repression of *AMIDASE 1* (*AMI1*) and the therewith coupled accumulation of IAM provide a molecular link that connects auxin and ABA biosynthesis pathways. In this way, plants are assumed to be able to marshal their energy resources and to fine-tune their growth with adequate responses to abiotic stress conditions. Moreover, another study has shown that ABA has a repressive effect on lateral root formation through the crosstalk with auxin in maize (Lu et al., 2019). It is assumed that the ABA receptor PYL9 enhances the transcription of *MYB77* (Xing et al., 2016), which is an interactor of ARF7 (Shkolnik-Inbar and Bar-Zvi, 2010). The latter is a key determinant of lateral root formation. In this manner, PYL9 can exert a direct impact on lateral root formation through transcriptional control of *MYB77*.

The myeloblastosis (MYB) transcription factor (TF) superfamily is large and functionally extremely diverse. Members of the MYB TF family share the conserved MYB DNA-binding domain, which generally consists of up to four imperfect amino acid sequence repeats (R) that span approx. 52 amino acids and form three α-helices. The second and third helix of each repeat fold into a helix-turn-helix (HTH) motif (Ogata et al., 1996). Regarding the number of MYB domains in the conserved N-terminal DNA binding domain, MYB factors can be divided into four classes: 1R, R2R3, 3R, and 4R-MYB proteins (Stracke et al., 2001; Dubos et al., 2010). Notably, the R2R3-MYB TF class is specific to the plant kingdom and the most abundant class of MYB factors in plants. As an example, the MYB superfamily in *Arabidopsis thaliana* is composed of only five 3R-MYB proteins and about 190 R2R3-MYB factors. The C-terminal regions of MYB family proteins are less conserved and contain a highly diverse modulator region that is responsible for the regulatory activity of the TFs. MYBs regulate a wide variety of biological processes in plants, ranging from their roles in controlling plant development through cell proliferation to their critical roles in diverse plant stress responses (Ambawat et al., 2013).

In this study, we present a detailed analysis of the role of MYB74. The *MYB74* gene has been identified as a downstream target of the auxin biosynthesis intermediate IAM. MYB74 clusters together with MYB41, MYB49, and MYB102 into subgroup 11 of Arabidopsis R2R3-MYB proteins (Kranz et al., 1998). While MYB41 has been identified as a negative regulator of transcriptional responses to osmotic stress (Lippold et al., 2009), MYB102 has been associated with osmotic stress responses and wound signaling pathways (Denekamp and Smeekens, 2003; De Vos et al., 2006). MYB49 was recently found to be involved in the modulation of salt tolerance, cuticle formation, and antioxidant defense (Zhang et al., 2020). As with most of the other members of subgroup 11, MYB74 has so far not been studied comprehensively. A previous study reports on the broad but weak expression of *MYB74* in Arabidopsis. Moreover, the work highlights the transcriptional activation of *MYB74* under salt stress and demonstrates a negative correlation of *MYB74* promoter DNA methylation with its induction under these conditions (Xu et al., 2015). However, the molecular processes downstream of MYB74 are largely unknown. Here, we provide evidence that elevated expression of *MYB74* results in substantial growth retardation, an effect that has also been observed for 35S-MYB41 mutants (Lippold et al., 2009). Furthermore, the conditional overexpression of *MYB74* affects the expression of a considerable number of abiotic stress-related genes and contributes to the modulation of the response to osmotic stress. The results presented here unveil a hitherto unknown molecular link between auxin biosynthesis and the induction of abiotic stress pathways in Arabidopsis. Moreover, our results consolidate the role of IAM as an important small signaling molecule that contributes to the coordination of the trade-off between growth and tolerance to osmotic stress, in part through the transcriptional control of *MYB74*.

## Materials and Methods

### Plant Material and Growth Conditions

Along with the reference genotype *A. thaliana* Col-0 (stock N1092), the following Arabidopsis mutant lines for *MYB74* (At4g05100) and *AMI1* (At1g08980) were used in this study: the *myb74* (SALK_073544C) T-DNA insertion line, the conditional overexpression lines MYB74oe-1 (TPT_4.05100.1D) and MYB74oe-2 (TPT_4.05100.1H) from the TRANSPLANTA collection (Coego et al., 2014), *ami1-2* and AMI1ind-2 (Pérez-Alonso et al., 2021a). Moreover, we used the abscisic acid (ABA) biosynthesis and signaling mutants *aba3-1* (Léon-Kloosterziel et al., 1996) and *abi5-7* (Nambara et al., 2002), respectively. The T-DNA insertion mutant for *MYB74* from the SALK collection was genotyped as described elsewhere (Alonso et al., 2003). The primers used for genotyping are given in Supplementary Table S1. After stratification (2 days, 4°C), plants were either grown sterilely on solidified 0.5X MS medium supplemented with 1% (w/v) sucrose (Murashige and Skoog, 1962) or on a mixture of peat and vermiculite (3:1). Plant growth was performed under environmentally controlled short-day conditions (8 h light at 24°C, 16 h darkness at 20°C, 105 μmol photons m^−2^ s^−1^ photosynthetically active radiation). The expression of the transgene in the conditional *MYB74* overexpression mutants MYB74oe-1 and MYB74oe-2, as well as in AMI1ind was induced by adding 10 μM ß-estradiol to the growth medium. In all experiments that employed these lines, corresponding wt control plants were grown on medium supplemented with equal amounts of ß-estradiol. To study osmotic stress responses, the plants were grown on media that were additionally supplemented with 300 μM mannitol. The investigation of heat stress tolerance was performed according to Hsieh et al. (2013). In brief, 24 seeds per genotype were sown on corresponding 0.5X MS media in sealed 90-mm Petri dishes. After the seeds were grown for 6 days under control conditions, they were subjected to a heat-shock treatment (30 min at 42°C). Thereafter, the plants were returned to control conditions and the survival rate was analyzed after 1 week of recuperation.

### Plant Hormone Analysis

The contents of ABA, IAA and IAM in 50 and 100 mg of 10 days old sterilely grown Arabidopsis seedlings, respectively, have been analyzed using mass spectrometry. While ABA was assessed by GC–MS/MS analysis according to Pérez-Alonso et al. (2021b), IAA and IAM were quantified by LC–MS/MS as previously described (Pérez-Alonso et al., 2021a). The content of each analyzed metabolite in wild-type Arabidopsis (Col-0) was set to 1. The metabolite contents in the MYB74oe lines were presented in reference to the wild-type values. All mass spectrometric analyses have been conducted in triplicate.

### Germination Assay

To analyze the impact of the conditional overexpression on seed germination, 50 seeds of each tested genotype were sown on Protran^®^ nitrocellulose membranes (Whatman) on MS medium supplemented with 10 μM ß-estradiol. The seeds were kept in a growth chamber under previously described standard conditions. The germination process of the different genotypes was periodically monitored under a Leica MZ10F stereomicroscope. To ensure equal conditions, all seeds were harvested at the same time, when the entire siliques had browned. The experiment was carried out in three biological replicates.

### Analysis of Stomatal Aperture

Whole leaves from four to 6 weeks-old Arabidopsis plants grown together under control conditions were harvested and the epidermis rapidly peeled. The abaxial epidermis peels were then placed on cover slips. The microscopic observation was done on a Zeiss LSM 880 (Carl Zeiss, Jena; Germany) microscope. The stomatal aperture was analyzed by measuring the quotient of the stomatal width and length using the Fiji image processing software (Schindelin et al., 2012). The experiments were repeated at least three independent times (*n* = 25).

### Modeling of the MYB74 and MYB102 Protein Structure

The three-dimensional structures of MYB74 and MYB102 were modeled by using a homology-based approach. The 2.15 Å crystal structure of MYB66 from *A. thaliana* [PDB: 6KKS] (Wang et al., 2020) and the 2.9 Å crystal structure of *Trichomonas vaginalis* MYB3 [PDB: 3ZQC] (Wei et al., 2012) deposited in the Research Collaboratory for Structural Bioinformatics (RCSB) Protein Data Base were used as reference structures. The different structural models were generated by using both the Phyre^2^ protein fold recognition server (Kelley et al., 2015) and the I-TASSER protein structure prediction server (Yang et al., 2015). The structural comparison of the obtained models for MYB74 and MYB102 was performed using PyMOL v2.5.0^1^ and the InterPro^2^ protein classification tool.

### RNA Isolation and Gene Expression Analysis by qRT-PCR

For each genotype and condition, 100 mg of plant tissue of seven to 10 days-old sterilely grown seedlings were harvested for total RNA extraction as previously described (Oñate-Sánchez and Vicente-Carbajosa, 2008). First strand synthesis was conducted using M-MLV reverse transcriptase and oligo(dT)_15_ primer, following the instructions of the manufacturer (Promega). Two nanograms of cDNA were used as template in each qRT-PCR. cDNA amplification was performed using the FastStart SYBR Green Master solution (Roche Diagnostics) and a Lightcycler 480 Real-Time PCR system (Roche Diagnostics), according to the supplier’s instructions. The relative transcript quantification was calculated employing the comparative 2^−∆∆CT^ method (Livak and Schmittgen, 2001). As reference genes, we used *APT1* (At1g27450) and *GAPC2* (At1g13440; Czechowski et al., 2005; Jost et al., 2007). The quantitative gene expression analysis and metabolite induction studies were carried out as previous described (Pérez-Alonso et al., 2021a), using three biological replicates. In addition, three technical replicates per biological replicate were analyzed. See Supplementary Table S1 for primer sequences.

### RNA-seq Analysis

In this study, we performed two genome-wide expression studies employing mRNA sequencing (mRNA-seq). First, total RNAs from 14 days-old wt, *ami1*, and AMI1ind seedlings treated with methanol (0.5% v/v, mock) or 100 μM indole-3-acetamide (IAM) for 3 h were extracted as described above and quantified using a Nanodrop ND-1000^®^ UV/Vis spectrophotometer (ThermoFisher). Overall RNA quality was additionally checked on a Bioanalyzer 2100 (Agilent) by the CNB Genomics Service (Madrid). Library construction and RNA sequencing (50-nt single-end reads) was performed by the Beijing Genomics Institute (BGI, Hong Kong, China) on Illumina HiSeq^™^ 2000 machines. Basic data analysis, including raw data cleaning, alignment of the clean reads to the Arabidopsis reference genome, and the quantitative analysis of differential gene expression, was performed using the BGI RNA-seq pipeline of standard bioinformatics. Secondly, mRNA from 10 days-old *myb74*, MYB74oe-1, and wild-type seedlings were subjected to mRNA-seq analysis. Library construction and sequencing (150-nt paired-end reads) on Illumina NovaSeq^™^ 6000 platforms was performed by the Novogene Genomics Service (Cambridge, United Kingdom). The Novogene Genomics Service also provided basic data analysis applying their RNAseq pipeline.

To analyze differentially expressed genes, Vulcano and Venn plots have been generated using the VolcaNoseR^3^ and Venn^4^ online tools, respectively. For the gene ontology (GO) enrichment analysis we used either the ClueGO application in Cytoscape or the g:Profiler online tool.^5^

### Statistical Analysis

The statistical assessment of the data was performed using the JASP v0.16.1 software.^6^ Student’s *t*-test was employed to compare two means, while two-way analysis of variance (ANOVA), followed by Tukey–Kramer’s multiple comparison test, was used for multiple mean comparison. The applied statistical analysis for each experiment is given in the corresponding figure legends. Results were considered significant when the value of *p* < 0.05.

## Results

### mRNA-Sequencing Reveals a Set of IAM-Responsive Genes in Arabidopsis

**Figure 1:**
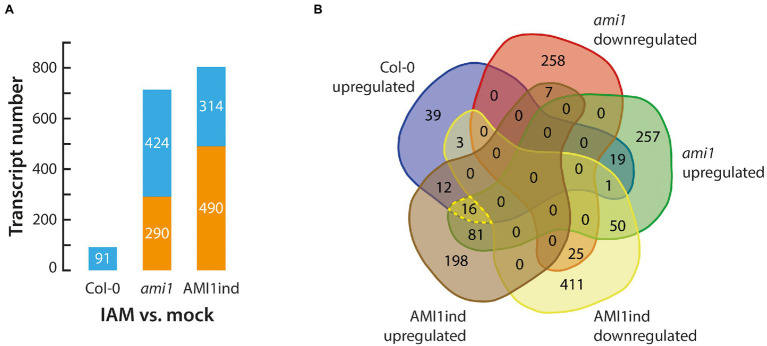
Gene expression analysis of IAM treated plants. **(A)** DEG statistics for 14 days-old Col-0 wild-type and *ami1* as well as AMI1ind mutants treated for 3 h with 100 μM IAM. **(B)** Venn diagram of significantly up and downregulated genes in the investigated genotypes. The group of commonly induced genes among the three genotypes is highlighted by a yellow dashed line.

IAM is an auxin precursor widely distributed in the plant kingdom (Sugawara et al., 2009; Sánchez-Parra et al., 2014). It is converted into IAA by the virtue of specific IAM hydrolases, including AMIDASE 1 (AMI1; Pollmann et al., 2003), and IAM HYDROLASE 1 (IAMH1) as well as IAM HYDROLASE 2 (IAMH2; Gao et al., 2020) from *A. thaliana*. Previous studies associated the accumulation of IAM with the induction of plant stress responses (Pérez-Alonso et al., 2021a). To gain insight into the biological processes and molecular mechanisms transcriptionally activated by high IAM levels, we profiled the transcriptomes of 14-days old Arabidopsis wild-type, *ami1*, and AMI1ind plants treated with 100 μM IAM for 3 h in comparison to mock-treated wild-type control plants to trigger strong transcriptional responses and provide sufficient substrate for the overexpressed amidase in the AMI1ind plants. As anticipated, given the already reported significant transcriptomic differences between the *AMI1* loss- and gain-of-function lines (Pérez-Alonso et al., 2021a), we identified significant transcriptional responses when the plants were treated with IAM (Figure 1A; Supplementary Table S2). Interestingly, the responses to IAM were substantially elevated in mutant plants with a manipulated expression of *AMI1* compared to the wild type. This confirms our previous observation that the alteration of *AMI1* expression provokes considerable changes in the transcriptomic profiles of the corresponding plants. As shown in Figure 1B, the Venn analysis of differentially expressed genes (DEGs), which was employed to prioritize candidates among the differentially expressed groups in the different genotypes, revealed a small group of 16 DEGs consistently induced under all tested conditions (Table 1). The consistent transcriptional activation of these genes under all tested conditions suggested a fast and presumably direct response to IAM. The remaining mRNA-seq data confirmed the notion of an induction of plant stress responses, elicited by the exogenous application of IAM and the genetically manipulated expression of *AMI1* (Moya-Cuevas et al., 2021). A KEGG pathway analysis, using the identified 16 genes as query, provided evidence for the overrepresentation of genes related with the galactose metabolism pathway. Out of the 16 genes the two galactinol synthase genes *GolS2* and *GolS3* belong to this pathway. Interestingly, galactinol synthases seem to be involved in conferring drought tolerance to plants and desiccation tolerance to seeds (Himuro et al., 2014; Lang et al., 2017), which links these genes with the abiotic stress responses triggered by elevated IAM contents. Next, we performed a GO analysis to identify biological processes enriched among the 16 genes. Fourteen of the sixteen genes could be associated to GO terms, and two GO term classifications, response to abiotic stimulus and response to abscisic acid, appeared to be significantly enriched (FDR < 0.05). The groups contained the genes *MYB102*, *GolS2*, *HAI1*, *COR15A* and *MYB74*, *AOC2*, *GolS2*, *GolS3*, *COR15A*, respectively (Supplementary Table S2). Our results indicated that a small number of genes that are related with abiotic stress responses show a considerable and relatively quick transcriptional response to IAM, even in conditional *AMI1* over-expressing mutants that convert exogenously applied IAM more rapidly to IAA.

**Table 1:** List of genes that were transcriptionally activated (IAM vs. control) by a short-term (3 h) treatment with 100 μM IAM in wild-type (Col-0) plants, the *AMI1* knockout (*ami1*) line, and the conditional AMI1 overexpression mutant (AMI1ind).

Gene ID	Gene name	Differential expression (*log*_2_FC)		
		Col-0	*ami1*	AMI1ind
At1g05100	*MAPKKK18*	1.07	1.63	1.39
At1g09350	*GolS3*	2.03	2.15	2.27
At1g21790		1.01	1.82	1.63
At1g53470	*MSL4*	1.13	1.92	2.02
At1g56600	*GolS2*	1.10	1.60	1.76
At2g36750	*UGT73C1*	1.01	1.68	1.62
At2g39980		1.04	1.24	1.08
At2g42540	*COR15A*	3.18	1.39	2.46
At3g25770	*AOC2*	1.33	1.39	1.03
At3g55500	*EXP16*	1.23	1.33	1.96
At4g05100	*MYB74*	1.44	1.34	1.18
At4g21440	*MYB102*	1.70	3.21	2.23
At4g33930		1.12	1.31	1.27
At5g10410	*PICALM10B*	2.05	1.73	2.02
At5g45950	*GGL28*	1.15	1.82	1.37
At5g59220	*HAI1*	1.35	2.23	2.19

All identified genes showed a significant differential expression (q-value_._ < 0.05).

### MYB74 and MYB102 Are Structurally Different

**Figure 2:**
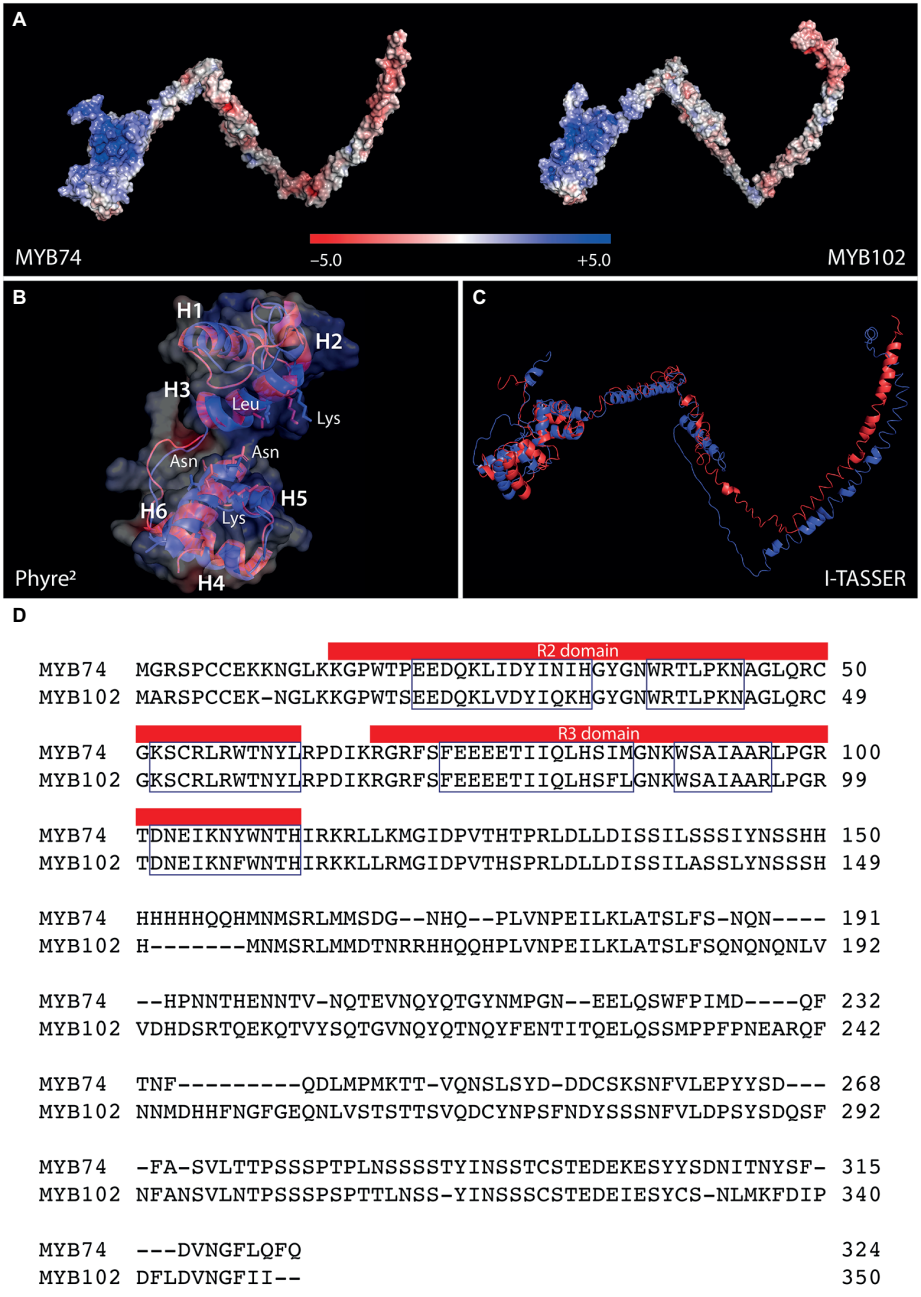
Structural comparison of Arabidopsis MYB74 and MYB102. **(A)** Electrostatic potential of MYB74 (left) and MYB102 (right). The color code for the estimated electrostatic potential is provided. **(B)** Superimposition of the conserved N-terminally located DNA binding domain. The three helices of the R2 (H1-H3) and the R3 domain (H4-H6) and the amino acids assumed to facilitate the protein-DNA binding are indicated. **(C)** Overlay of the complete three-dimensional structure models for MYB74 (red) and MYB102 (blue). The models have been inferred using the I-TASSER **(A,C)** and Phyre^2^ **(B)** structural prediction server. **(D)** Sequence alignment of MYB74 and MYB102. The R2 and R3 sequence repeats are highlighted in the figure and the three helices of each repeat are indicated by boxes. Domain identification has been carried out using the InterPro classification tool.

Among the identified target genes, the two members of the R2R3-MYB TF family, MYB74 and MYB102, arouse our particular interest. MYB102 has previously been studied in closer detail and has been associated with wounding, osmotic stress responses, and the induction of ethylene biosynthesis (Denekamp and Smeekens, 2003; De Vos et al., 2006; Zhu et al., 2018), while the physiological function of MYB74 remained largely elusive, except its response to salt stress and its putative connection with ABA signaling (Xu et al., 2015; Huang et al., 2018a). Regarding their primary amino acid sequences, the two MYB factors share 65.7% identity. To gain further insight into the structural intricacies of the two TFs, we built homology-based protein models using the I-TASSER (Yang et al., 2015) and Phyre^2^ (Kelley et al., 2015) interfaces with the *A. thaliana* MYB66 [6kks] (Wang et al., 2020) and the *Trichomonas vaginalis* MYB3 [3zqc] (Wei et al., 2012) structures as template. The models revealed a substantial structural similarity of the two factors in their conserved N-terminally located DNA binding domains, which are recognizable by the positive surface potential (Figure 2A). The DNA binding domain of R2R3-MYB factors is composed of two adjacent imperfect amino acid sequence repeats (R), each consisting of three α-helices (Figures 2B,D). The nearly perfect superimposition of the DNA binding (DB) domains (Figure 2B) and the largely identical primary amino acid sequence in the N-terminal region (Figure 2D) demonstrated the high structural conservation of the two factors in their DB domains. Irrespective from minor displacements, also the anticipated amino acid residues in helix 3 (MYB74: Lys52 and Leu56; MYB102: Lys51 and Leu55) and helix 6 (MYB74: Asn103, Lys106, and Asn107; MYB102: Asn102, Lys105, and Asn106), which are assumed to be responsible for protein-DNA interaction (Wang et al., 2020), appeared structurally conserved. However, as shown in Figure 2C, the C-terminal modulator regions, which are responsible for the regulatory properties of the proteins, are clearly divergent. These non-MYB regions contain extensive intrinsically disordered regions (IDRs) that do not fold into stable globular structures, but mediate the interactions with many different signaling molecules and proteins through the formation of a large portfolio of transient structures (Millard et al., 2019). In summary, the *in-silico* structural modeling confirmed shared DNA-binding properties, but do not support the notion of overlapping regulatory functions, because the IDRs of MYB74 and MYB102 are most likely too different to cover similar regulatory purposes.

### IAM-Induced *MYB74* Expression Is Independent of ABA

**Figure 3:**
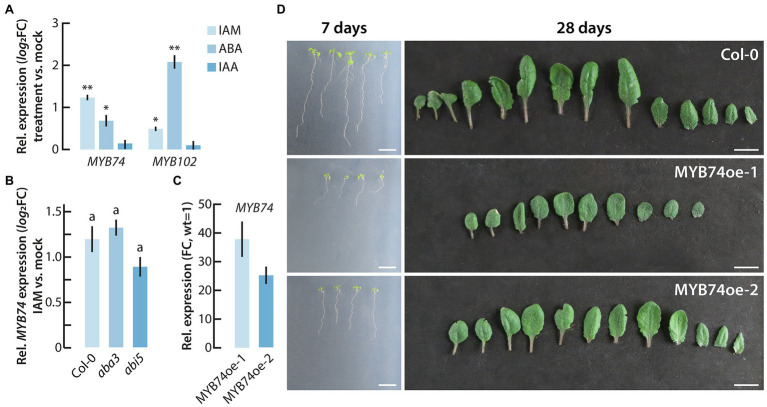
Phenotypic analysis of conditional *MYB74* overexpressor plants. **(A)** Transcriptional activation of *MYB74* and *MYB102* after the short-term treatment of wild-type Arabidopsis seedlings with either 20 μM IAM, IAA, or ABA for 2 h. The data show the means with their corresponding SE (*n* = 9). Student’s *t*-test: ^*^p ≤ 0.05; ^**^p ≤ 0.01. **(B)** Differential expression of *MYB74* in wild-type seedlings (Col-0), and the ABA mutants *aba3* and *abi5*. The plot depicts the relative *MYB74* expression in IAM versus mock treated seedlings. The bar plots represent means ±SE (*n* = 9). Different letters indicate significant differences between the means of the compared genotypes assessed by ANOVA and a Tukey–Kramer *post hoc* test (*p* ≤ 0.05). **(C)** Expression level of *MYB74* in the two conditional MYB74 overexpressor lines, MYB74oe-1 and MYB74oe-2. The expression is shown relative to the gene expression level in wild-type seedlings, which have been set to 1. **(D)** Phenotypic appearance of wild-type (Col-0), MYB74oe-1, and MYB74oe-2 plants grown either for 7 days on sterile 0.5X MS plates (left) or for 28 days on soil (right). Scale bars = 1 cm.

Next, we performed qPCR analysis of *MYB74* and *MYB102* expression in IAM, ABA, IAA, and mock treated wild-type Arabidopsis seedlings. As shown in Figure 3A, *MYB74* showed a stronger response to IAM compared to *MYB102*, while the latter responded more strongly to ABA. This finding is consistent with our previous study in which we reported the differential expression of several TFs in the *ami1* mutant, including *MYB74* and *MYB102* (Pérez-Alonso et al., 2021a). Intriguingly, neither *MYB74* nor *MYB102* were transcriptionally activated when wild-type plants were treated with IAA. For this reason, a partially IAA-dependent regulation through the conversion of IAM to IAA by the IAM hydrolases AMI1, IAMH1, and IAMH2 can likely be ruled out. Given the stronger response of *MYB74* to IAM and its responsiveness to short- and long-term osmotic stress (Watanabe et al., 2018), which further connects the factor with the *ami1* osmotic stress phenotype (Pérez-Alonso et al., 2021a), we focused our attention in the following on MYB74.

While the knockout of *IAMH1* and *IAMH2* has not been reported to translate into altered IAA and IAM levels under control conditions (Gao et al., 2020), the functional impairment of AMI1 in the *ami1* mutant alleles is known to significantly increase both endogenous IAM and ABA contents (Pérez-Alonso et al., 2021a). Therefore, the transcriptional response of *MYB74* could be triggered directly by IAM or proceed *via* the induction of ABA biosynthesis. To further our understanding of the transcriptional regulation of *MYB74*, we quantified the expression of *MYB74* after IAM and mock treatment in wt, *aba3*, and *abi5* seedlings by qPCR. Given that *aba3* is an ABA deficient and *abi5* an ABA insensitive mutant (Léon-Kloosterziel et al., 1996; Finkelstein and Lynch, 2000), we were able to address the question whether the induction of *MYB74* expression is dependent on ABA. As shown in Figure 3B, we detected no significant difference in the response of *MYB74* toward the treatment with IAM in the ABA mutants. Notably, the *abi5* mutant showed a slightly lower induction of *MYB74* expression, but the determined difference to the wild-type control was not significant. Together with the missing transcriptional response of MYB74 toward IAA (Figure 3A), this result suggested that the response of *MYB74* towards IAM occurs largely uncoupled from the formation of ABA.

### MYB74 Negatively Affects Plant Growth

From a previous study (Xu et al., 2015), it is known that *MYB74* is constitutively expressed in the plant in low abundance. The highest levels of expression are detected in leaves and flowers. Furthermore, the authors provided evidence that overexpression of *MYB74* causes salt hypersensitivity during seed germination. To gain more insight into the physiological role of MYB74, we took a reverse genetics approach and performed a more detailed phenotypic analysis of *MYB74* mutants. As with the RNAi lines reported by Xu et al. (2015), the tested *myb74* T-DNA integration line showed only very moderate, if any, differences compared to the wild type, although the *MYB74* expression level in *myb74* was shown to be significantly reduced (Supplementary Figure S1). However, the inspection of two conditional *MYB74* overexpression lines, MYB74oe-1 and MYB74oe-2, which are characterized by a 38- and 25-fold overexpression of the transgene (Figure 3C), respectively, provided intriguing insight into the function of MYB74 as a negative plant growth regulator. As can be taken from Figure 3D, the two independent conditional overexpression lines showed a clear growth reduction when grown under control conditions. Both sterilely grown MYB74oe seedlings and soil grown MYB74oe plants showed considerable growth reductions, which could also be confirmed by the determination of fresh and dry weights of the mutants relative to wild-type plants (Supplementary Figure S2).

### MYB74 Controls Diverse Abiotic Stress Responses

**Figure 4:**
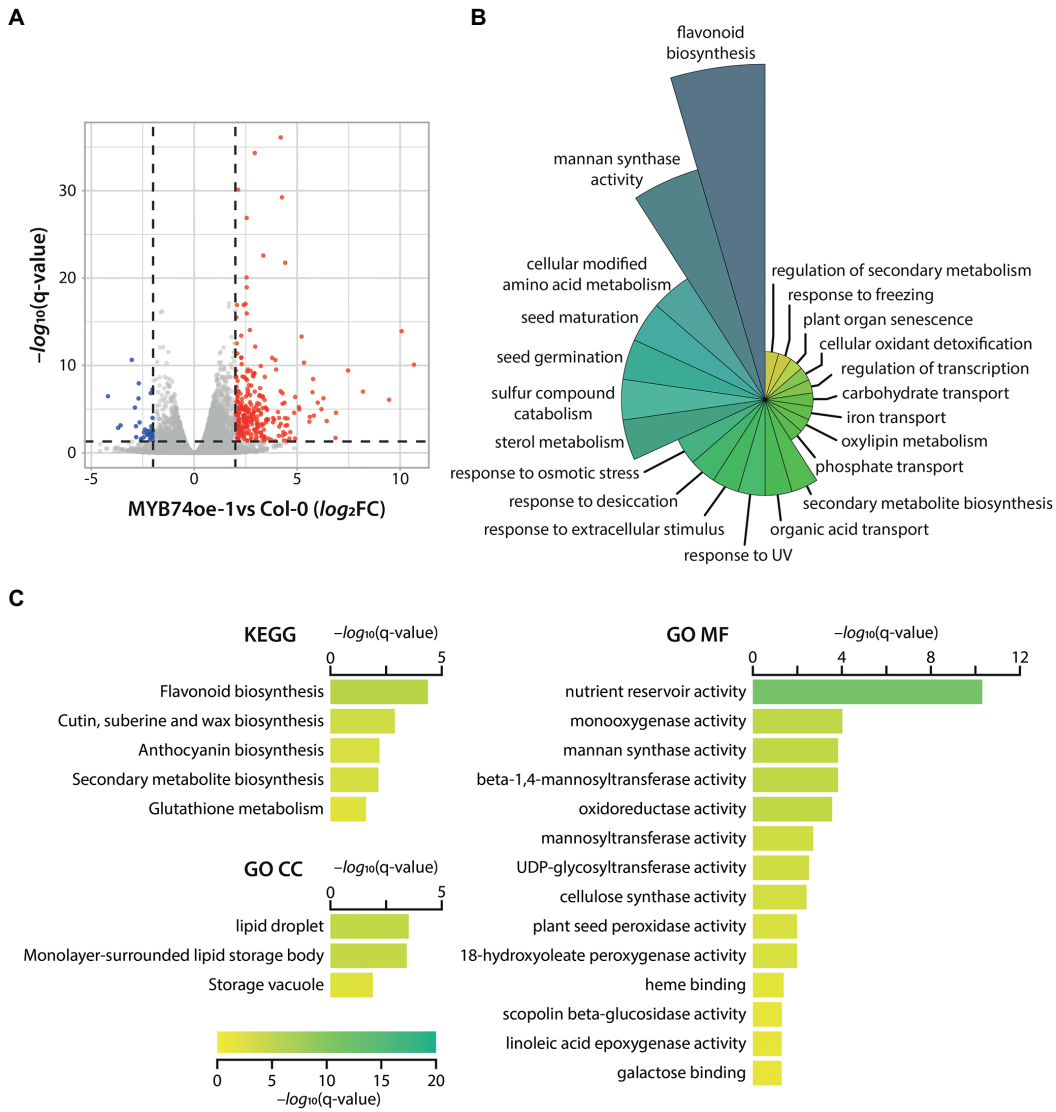
Transcriptomics analysis of differentially expressed genes (DEGs) in the transgenic MYB74oe-1 line. **(A)** Volcano plot of differentially expressed genes in MYB74oe-1 compared to wild-type (Col-0) seedlings. MYB74 acts as a transcriptional activator, as the majority of the DEGs appear to be induced (red dots), while only a small number of genes is repressed (blue dots). Genes with no significant changes (*log*_2_FC = ±1.75, q-value = 0.05) are show in grey. **(B)** Polar plot of biological process-related GO terms enriched in the identified DEGs in MYB74oe-1. **(C)** Bar plots of KEGG pathway (KEGG) and GO cellular compartment (GO CC) as well as molecular function (GO MF) enrichment analysis. The color and length of the bars reflect the significance of the different identified terms, indicated by the legend at the bottom [−*log*_10_(q-value)]. The graphs only show significantly enriched terms with a q-value <0.05.

With the aim to further characterize the physiological function of MYB74, the *myb74* T-DNA insertion line and MYB74oe-1 were subjected to mRNA-seq analysis, comparing their transcriptional profiles to that of Col-0 control plants (Supplementary Table S3). Consistent with the phenotypic analysis of the *myb74* mutant, the RNA-seq analysis of the knockout line provided evidence for only a very reduced number of DEGs. Overall, we found five repressed and three induced genes (threshold *log*_2_FC = ±1.25, q-value = 0.05), suggesting that MYB74 plays no critical role under control conditions. On the contrary, as shown in Figure 4A, the transcriptional profiling of MYB74oe-1 relative to the wild type disclosed a total of 355 induced and 67 repressed genes (threshold *log*_2_FC = ±1.75, q-value = 0.05). Interestingly, we found *MYB102* to be significantly induced (*log*_2_FC = 2.71) in the conditionally *MYB74* overexpressing line. This is indicative for a secondary transcriptional activation of *MYB102* through the elevated expression of *MYB74* in the transgenic plants.

Taking into consideration that *MYB74* is induced by the abiotic stress-triggered increases in both IAM and ABA levels, it must be concluded that MYB74 is involved in the integration of stress stimuli and the reprograming of the transcriptome to adequately respond to the perceived signals. Functional gene enrichment analyses using the g:Profiler (Raudvere et al., 2019), ClueGO (Bindea et al., 2009), and Metascape (Zhou et al., 2019) tools revealed, among other GO terms and KEGG pathways, the overrepresentation of osmotic stress, water deprivation, desiccation, and seed maturation, development, as well as germination-related gene sets (Figures 4B,C; Supplementary Table S3). Particularly the abiotic stress-related GO classifications showed a considerable number of shared genes, including *ERF53* (At2g20880), *PXG3* (At2g33380), *COR15A* (At2g42540), *LTP4* (At5g52300), *LTI65* (At5g52300), WSD1 (At5g37300), *GolS2* (At1g56600), *MAPKKK18* (At1g05100), and *RD29A* (At5g52310), some of which (*COR15A*, *GolS2*, and *MAPKKK18*) have already been identified as primary IAM target genes. Notably, *ERF53* was also found to be induced in the *ami1* null mutant (Pérez-Alonso et al., 2021a). With respect to water deprivation response-related genes, we identified *ANAC019* (At1g52890), *ANAC055* (At3g15500), and *ANAC072* (At4g27410) as particularly interesting group members. These plant specific NAC (petunia NAM and *Arabidopsis* ATAF1, ATAF2, and CUC2) TFs have previously been associated with stress responses and the regulation of stomatal aperture (Hickman et al., 2013; Gimenez-Ibanez et al., 2017).

**Figure 5:**
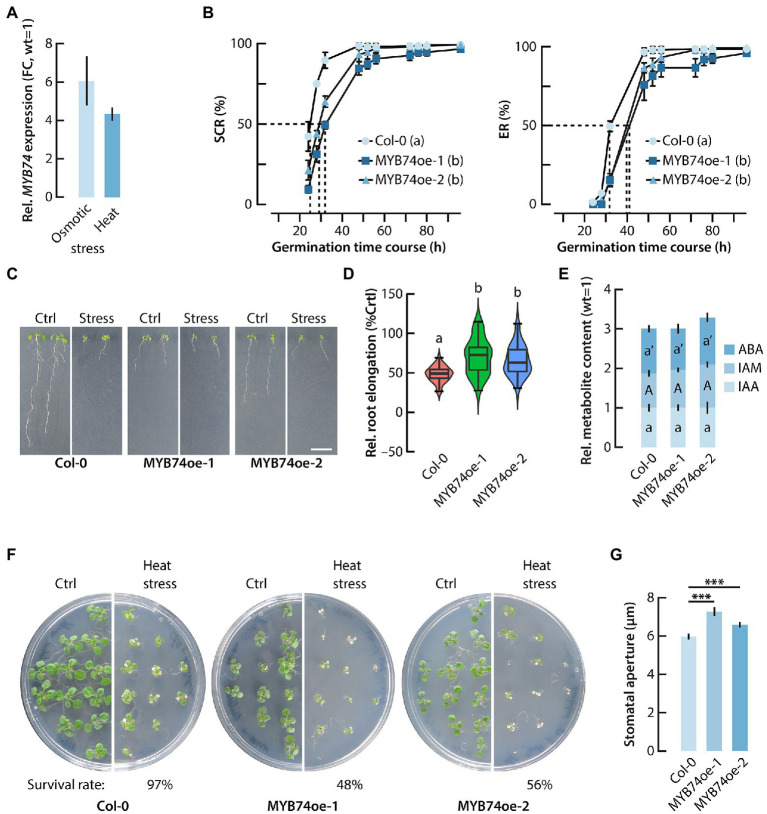
Functional characterization of MYB74oe lines. **(A)** qPCR analysis of the transcriptional activation of *MYB74* by heat and osmotic stress conditions. Gene expression is given relative to the *MYB74* expression in untreated control plants, which has been set to 1. **(B)** Seed germination kinetics in wild-type Arabidopsis (Col-0) and the two conditionally MYB74 overexpressing lines. Seed coat rupture (SCR) and endosperm rupture (ER) time course. Different letters indicate significant differences (*p* < 0.05) in germination of the tested genotypes at t_50_(h), indicated by the dashed lines. Two-way ANOVA analysis with a subsequent Tukey–Kramer *post hoc* test was employed to statistically assess the data. **(C)** Phenotypic analysis of primary root growth under control (Ctrl) and osmotic stress condition (Stress). To apply osmotic stress, young seedlings were grown on plates containing 300 mM mannitol. Scale bar = 1 cm. **(D)** Violine plot showing the relative primary root elongation response of 4 days-old seedlings that were transferred to plates containing 300 mM mannitol and grown for another 7 days. The primary root elongation of each genotype grown under control conditions was set to 100%. The boxes and whiskers display the median, quartiles, and extremes of the compared datasets (*n* = 20). The colored regions indicate the density distribution of the samples in each genotype and condition. Different letters refer to significant differences between the means of the compared datasets analyzed by ANOVA and a Tukey–Kramer *post hoc* test (*p* ≤ 0.05). **(E)** Stacked bar plots representing the relative quantification of endogenous IAA, IAM, and ABA contents in wild-type Arabidopsis and the two MYB74oe lines. The content of each metabolite in wild-type seedlings (Col-0) was set to 1. The metabolite levels in the MYB74oe lines were expressed in reference to the wild-type levels. The stacks show mean values ±SE (*n* = 3). The same letters for each metabolite in the different genotypes refer to a lack of significant differences between the means of the compared datasets analyzed by ANOVA and a Tukey–Kramer *post hoc* test (*p* ≤ 0.05). **(F)** Survival rate of Col-0 and MYB74oe seedlings 7 days after a heat shock treatment (42°C, 30 min). The conditional overexpression of *MYB74* reduced the survival rate of the seedlings considerably. **(G)** Comparison of the stomatal aperture of wild-type (Col-0) and MYB74oe lines under control conditions. The overexpression of *MYB74* significantly increases stomatal aperture. Student’s *t*-test: ^***^p ≤ 0.001.

Apart from an involvement in steering flavonoid biosynthesis, MYB74 seems to contribute to the control of the expression of a substantial number of cellulose synthase-like (*CSL*) genes, such as *CSLA1* (At4g16590), *CSLB1* (At2g32610), *CSLB2* (At2g32620), *CSLG1* (At4g24010), *CSLG2* (At4g24000), and *CSLG3* (At4g23990). The expression of genes related with the formation of cellulose and hemicellulose largely determines the biomechanical properties of plant secondary cell walls. Intriguingly, previous studies suggest a role for CSLs in salt tolerance (Zang et al., 2019), root hair morphogenesis (Favery et al., 2001), and organ size determination by altering cell division (Li et al., 2018b). Overall, the significant induction of genes related with osmotic stress protection and damage-repair pathways, including late embryogenesis abundant (*LEA*) and *LEA*-like genes as well as genes coding for proteins with similar characteristics such as the cold-regulated (COR)/responsive to desiccation (RD)/low temperature induced (LTI) proteins, must be highlighted. It remains to be noted that, in comparison with wild-type seedlings, the overexpression of *MYB74* in the MYB74oe lines does not interfere with the endogenous levels of IAA, IAM, and ABA (Figure 5E).

### MYB74 Overexpressing Plants Show Increased Osmotic Stress Tolerance

Addressing the previously described considerable transcriptional regulation of *MYB74* by abiotic stress cues (Kilian et al., 2007), we confirmed the response of *MYB74* to heat and osmotic stress conditions by qPCR analysis (Figure 5A). Taking the previously reported enrichment of abiotic stress- and seed development as well as germination-related genes in the conditional MYB74 overexpression line into account, we further investigated seed germination, and both heat and osmotic stress tolerance of the MYB74oe lines. First, we analyzed the effect of the conditional *MYB74* overexpression on the germination process. As shown in Figure 5B, the induction of *MYB74* through the addition of ß-estradiol to the medium clearly interfered with the germination process. The two MYB74oe lines exhibited a significant delay in seed germination, both in terms of seed coat rupture (SCR), defined as the visible vertical opening of the seed coat provoked by the mechanical force exerted by the embryo, and endosperm rupture (ER), defined as the radicle emergence through the seed coat (Iglesias-Fernández et al., 2013). Next, we characterized the primary root growth of MYB74oe lines in comparison to wild-type seedlings under osmotic stress conditions (Figure 5C). Apparently, the primary root length of the MYB74oe lines under control conditions was already significantly reduced (Figure 5C; Supplementary Figure S2). However, when the plants were first germinated on 0.5X MS for 4 days and then transferred to plates containing mannitol, the relative stress response, as defined by the observed primary root elongation, of the MYB74oe lines was clearly less pronounced. While wild-type seedlings showed a reduction of primary root elongation of 52%, the MYB74oe-1 and MYB74oe-2 seedlings displayed a reduction of only 29 and 34%, respectively (Figure 5D). Next, we assessed the response of the lines towards a short-term heat treatment. For this, plates with 24 six days-old seedlings were subjected to a heat shock (42°C, 30 min) and the survival rate of the seedlings was monitored after a seven-day long recovery phase. As shown in Figure 5F, the survival rate of MYB74oe-1 and MYB74oe-2 seedlings was drastically reduced, which suggested an impairment of the heat shock response (HSR). This promoted us to take a closer look at the expression of HSR-related genes. The mRNA-seq data revealed a significant repression of *HSFA7a* (At3g51910) and the induction of *HSFA6a* (At5g43840). Previous studies have characterized *HSFA6a* as an activator of stress-responsive genes in ABA-dependent signaling pathways and highlighted its involvement in a few other processes, including stomatal movement and water loss (Hwang et al., 2014; Wenjing et al., 2020). Interestingly, *HSFA6a* is not induced by heat, but only by high salt conditions and dehydration (Hwang et al., 2014). HSFA7a, on the contrary, acts in conjunction with HSFA2 in the control of HSR-related genes (Lin et al., 2018). Apart of the differential expression of *HSFA6a* and *HSFA7a*, the mRNA-seq results indicated no differential expression of other HSR-related genes, such as heat shock proteins (*HSPs*), *DREB2A*, or *MBF1c* (Jacob et al., 2017). For this reason, we decided to compare the stomatal aperture of MYB74oe-1 and MYB74oe-2 leaves with that of leaves from wild-type plants. A misregulation of the stomatal aperture could explain the reduced survival rate of the conditional *MYB74* overexpressor lines through increased water loss. Consistent with the observed reduction of the survival rate of MYB74oe-1 and MYB74oe-2 seedlings after a heat shock, we detected a significant increase of the stomatal aperture of the overexpressor lines (Figure 5G). Taken together, our results suggest a role for MYB74 as a negative plant growth regulator. In addition, it must be concluded that MYB74 contributes to the control of the osmotic stress response in Arabidopsis and interferes with stomatal closure.

## Discussion

### Transcriptional Insights From the Analysis of IAM Treated Plants

Previous studies unveiled a role for IAM as a putative new signaling molecule in plants, which is further supported by the fact that IAM and IAA regulate largely different subsets of genes, when comparing the transcriptional responses of IAM-treated *ami1* seedlings with those of IAA treated wild-type seedlings (Supplementary Figure S3). IAM has been demonstrated to trigger the expression of plant stress response-related genes, induce the formation of ABA, and cause a significant plant growth retardation (Moya-Cuevas et al., 2021; Sánchez-Parra et al., 2021; Pérez-Alonso et al., 2021a). The observed enhanced expression of *NCED3* after IAM application and in IAM accumulating *ami1* mutants indicate that the activation of ABA metabolism is responsible for the deregulation of a considerable number of stress-related genes. In addition, the specific IAM hydrolase *AMI1* from Arabidopsis has been shown to be transcriptionally regulated by several abiotic stress conditions, including drought, osmotic, and temperature stress (Lehmann et al., 2010; Pérez-Alonso et al., 2021a). However, many of the molecular mechanisms downstream of IAM, particularly those related with the observed growth retardation, remained largely undisclosed. Thus, further work was required to shed light on the biological processes activated by the stress-mediated accumulation of IAM in plants.

Transcriptomic analysis of IAM-treated wild-type and *AMI1* mutant plants identified a set of well-known stress-responsive genes (Figure 1; Table 1), including *MAPKKK18*, *COR15A*, and *HAI1*, which are part of the overall response to drought, cold, and heat stress (Maruyama et al., 2004; Fujita et al., 2009; Li et al., 2017). Furthermore, the experiment revealed the transcriptional activation of two MYB TFs, *MYB74*, and *MYB102*. The two MYB factors belong to the same R2R3-MYB factor subgroup 11 and share considerable primary amino acid sequence similarity (Supplementary Figure S4). Notably, neither *MYB74* nor *MYB102* displayed a significant transcriptional response toward IAA, which puts the conversion of IAM into IAA by IAM-specific hydrolases into perspective (Figure 3A). Although their N-terminal regions appeared to be highly conserved, the C-terminal IDRs of the two factors, which contain the response regulator domains, showed to be substantially divergent (Figure 2).

### Overexpression of *MYB74* Has a Detrimental Effect on Plant Growth

In general, MYB factors are expected to be involved in stress responses, but only a modest number has yet been characterized functionally. Interestingly, the four members of R2R3-MYB factor subgroup 11, comprising *MYB41*, *MYB49*, *MYB74*, and *MYB102*, share a common osmotic and salt stress responsiveness (Lippold et al., 2009). Their close phylogenetic relationship (Supplementary Figure S4) and their common transcriptional regulation point towards the existence of an intertwined regulatory MYB TF subnetwork that drives transcriptional reprogramming in Arabidopsis to mount appropriate abiotic stress responses.

The four subgroup 11 members are assumed to be transcriptionally controlled by ABA. However, our results point to an additional, ABA independent transcriptional regulation of *MYB74* expression by IAM (Figures 3A,B). Most interesting, however, was the observed growth retardation in conditional *MYB74* overexpressing mutants (Figures 3C,D, Figure 5B). A similar phenotype has been described for constitutively *MYB41* overexpressing transgenic plants (Cominelli et al., 2008; Lippold et al., 2009). MYB41 over-accumulating plants are characterized by increased rates of water loss and by substantially reduced cell sizes, but the underlying molecular mechanisms responsible for the phenotypic alterations remained largely unclear. It remains to be noted that the MYB74oe mutants tested in this study are also prone to increased water loss, as their stomatal aperture was shown to be significantly increased (Figure 5G). Such effect is remarkable for a gene that is normally induced in response to heat stress (Figure 5A). The transcriptional analysis of MYB74oe-1 compared to wild-type control plants provided no clear evidence for the existence of a molecular mechanism that could explain the pleiotropic growth phenotype of the mutant plants. A closer inspection of the differentially regulated transcription factors, however, revealed the deregulation of two additional MYB factors (Supplementary Table S3), *MYB11* and *MYB77*, that most likely contribute to the aberrant growth phenotype of the MYB74 accumulating lines. While the repression of *MYB77* is assumed to negatively affect lateral root formation (Shin et al., 2007), the transcriptional activation of *MYB11* is supposed to interfere with the overall proliferation activity of meristematic cells in Arabidopsis and, thereby, delay plant development (Petroni et al., 2008). However, to confirm and further develop these molecular relationships, much more work is required.

### Toward a Role for MYB74 in Orchestrating Plant Stress Responses

The transcriptional profiling data that we obtained for the *MYB74* mutants support our hypothesis that MYB74 has no crucial function under control conditions but is induced when the plants encounter abiotic stress. The considerably higher number of significantly induced genes relative to the number of repressed genes clearly suggests that this MYB TF plays a merely gene activating role (Figure 4A), which consequently translates into a negative effect on plant growth. Further enrichment analyses provided evidence for an involvement of MYB74 in the regulation of a broad number of abiotic stress response-related processes (Figures 4B,C). As already mentioned, previous studies suggested the induction of the subgroup 11 MYB TFs by heat, osmotic, and salt stress. For this reason, we decided to focus our interest on heat and osmotic stress responses in the characterization of the MYB74oe mutants, because *ami1* null mutants have also been reported to be involved in the osmotic stress response pathway (Pérez-Alonso et al., 2021a) and other abiotic stress responses (Lehmann et al., 2010). The significantly less pronounced relative primary root elongation inhibition observed in the MYB74oe lines speaks for an enhanced osmotic stress tolerance of the transgenic plants (Figure 5D). This notion is further supported by the substantial induction of *LEA*, *LEA-like*, *COR*, *RD*, and *LTI* genes, which are suggested to be involved in conferring an increased stress tolerance to plants through the protection of proteins from inactivation and aggregation and of membranes from disintegration under stress (Nakashima and Yamaguchi-Shinozaki, 2006). Moreover, the transcriptomics data highlighted the transcriptional activation of flavonoid biosynthesis-related genes (Figure 4B). Very recently, the accumulation of flavonoids has been shown to alleviate combined heat and salt stress in rice (Jan et al., 2021). Remarkably, the prolonged high-level expression of *MYB74* also linked the TF with processes that are not necessarily interwoven with abiotic stress responses. As an example, we found a series of induced genes that are associated with nutrient deficiency responses in Arabidopsis, including the sulfate-deficiency induced gene *SDI1* (At5g48850) and the response to low sulfur gene *LSU1* (At3g49580), as well as the phosphate deficiency-associated SPX domain genes *SPX1* (At5g20150) and *SPX3* (At2g45130), the *PHO1* sulfate transporter homolog gene *PHO1-H10* (At1g69480), and the phosphate-starvation induced inositol-3-phosphate synthase 2 gene *IPS2* (At2g22240). These data suggest that MYB74 may also be involved in the control of nutrient assimilation. However, to draw further conclusions about the presumably diverse functions of MYB74, additional metabolomics studies will be required.

**Figure 6:**
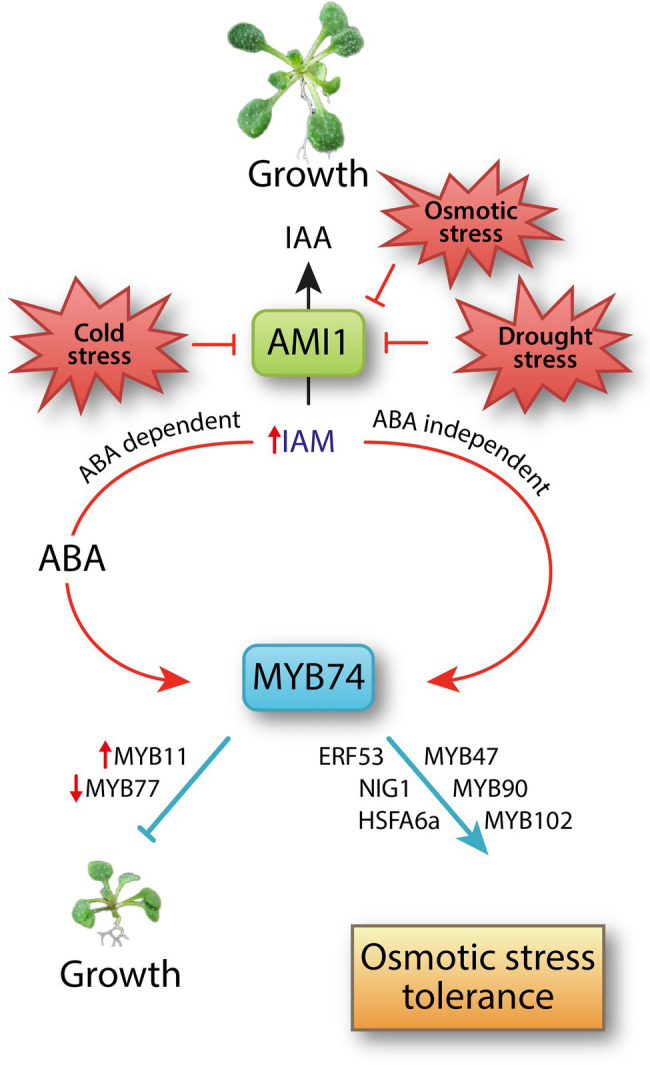
A model summarizing the IAM accumulation-mediated transcriptional activation of *MYB74*. Abiotic stresses, including osmotic stress, suppress the expression of *AMI1* (Perera et al., 2008; Lehmann et al., 2010), which translates into an accumulation of IAM. The auxin precursor IAM triggers ABA biosynthesis (Pérez-Alonso et al., 2021a). Here, we demonstrated an ABA dependent and an ABA independent transcriptional activation of abiotic stress-related TF *MYB74*. IAM directly induces the expression of *MYB74*. The IAM-mediated accumulation of MYB74 results in the transcriptional reprogramming of many osmotic stress-related genes, including further MYB factors, such as the dehydration stress memory gene *MYB47* (Ding et al., 2013) and the abiotic stress-related genes *MYB90* and *MYB102* (Denekamp and Smeekens, 2003; Li et al., 2018a), the ethylene response factor *ERF53* (Hsieh et al., 2013), the basic helix–loop–helix factor *NIG1* (Kim and Kim, 2006), and the ABA-responsive heat shock response factor *HSFA6a* (Hwang et al., 2014). Consequently, MYB74 is assumed to integrate ABA dependent and independent signals and to be involved in plant responses to osmotic stress. At the time, the accumulation of MYB74 suppresses plant growth considerably, possibly through the transcriptional activation of *MYB11*, which is known to be capable of delaying plant development (Petroni et al., 2008), and the repression of *MYB77* expression. MYB77 is involved in the modulation of auxin signal transduction and the control of lateral root formation (Shin et al., 2007).

As summarized in Figure 6, we demonstrated the involvement of an ABA dependent and an ABA independent signaling pathway that proceeds directly through the activation by IAM, in the transcriptional regulation of *MYB74*. Our transcriptiomics, physiological, and phenotypic analysis of *MYB74* mutants revealed a contribution of the MYB factor in the control of distinct cellular processes in Arabidopsis, including the positive regulation of osmotic stress tolerance. Moreover, our work uncovered a growth retarding effect through the prolonged overexpression of *MYB74*. Furthermore, it must be remarked that MYB74 seems to also interfere with the regulation of stomatal aperture, rendering the MYB74 over-accumulating substantially more susceptible to heat stress.

## Data Availability Statement

The authors acknowledge that the data presented in this study must be deposited and made publicly available in an acceptable repository, prior to publication. Frontiers cannot accept a manuscript that does not adhere to our open data policies.

## Author Contributions

SP and MW conceptualized the project and were responsible for acquiring the funding. PO-G, M-MP-A, AGO-V, BS-P, JL-M, and SP performed the experiments. PO-G, M-MP-A, AGO-V, BS-P, JL-M, MW, and SP analyzed and interpreted the data. SP wrote the paper with significant input from all other authors. All authors contributed to the article and approved the submitted version.

## Funding

PO-G is particularly grateful to all members of the Ludwig-Müller lab for their hospitality and to the Universidad Politécnica de Madrid, that financed the short-term stay in the Technische Universität Dresden, Germany, through its “Programa Propio UPM 2021” mobility program. This research was supported by grants BFU2017-82826-R and PID2020-119441RB-100 to SP funded by MCIN/AEI/10.13039/501100011033 and as appropriate, by “ERDF A way of making Europe,” by the “European Union” or by the “European Union NextGenerationEU/PRTR.”

## Conflict of Interest

The authors declare that the research was conducted in the absence of any commercial or financial relationships that could be construed as a potential conflict of interest.

## Publisher’s Note

All claims expressed in this article are solely those of the authors and do not necessarily represent those of their affiliated organizations, or those of the publisher, the editors and the reviewers. Any product that may be evaluated in this article, or claim that may be made by its manufacturer, is not guaranteed or endorsed by the publisher.

## Footnotes

^1^ https://pymol.org/

^2^ https://www.ebi.ac.uk/interpro/

^3^ https://huygens.science.uva.nl/VolcaNoseR2/

^4^ http://bioinformatics.psb.ugent.be/webtools/Venn/

^5^ https://biit.cs.ut.ee/gprofiler/gost

^6^ https://jasp-stats.org/
