# Supplementary material for: The Indole-3-Acetamide-Induced Arabidopsis Transcription Factor MYB74 Decreases Plant Growth and Contributes to the Control of Osmotic Stress Responses
Source: Front Plant Sci. 2022 Jun 22;13:928386. doi: 10.3389/fpls.2022.928386 (PMC9257185; doi:10.3389/fpls.2022.928386)
Supplement: Supplementary file 4 [file Data_Sheet_1.pdf]

Figure S1:

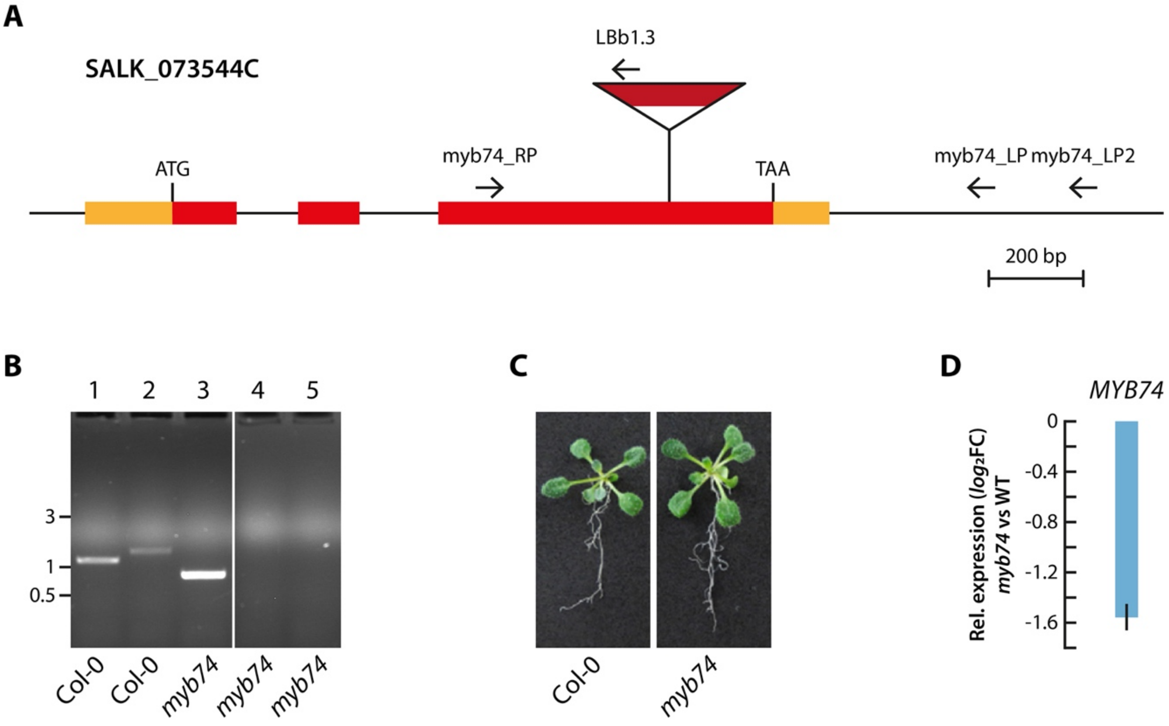

Figure S1. Genotyping of the *myb74* T-DNA insertion line. **A.** Diagram showing the gene structure of *MYB74* (At4g05100). The location of the T-DNA insertion in exon 3 has been confirmed by sequencing. The arrows indicate the position and orientation of the primers used for genotyping. ATG, start codon; TAA, stop codon. **B.** PCR zygosity analysis of *myb74*. One T-DNA and three gene specific primers were used in the following combinations for genotyping: (1) myb74_RP and myb74_LP and (2) myb74_RP and myb74_LP2, flanking the T-DNA insertion. (3) myb74_RP and LBb3.1 and (4) myb74_LP and LBb3.1, comprising the T-DNA left boarder primer and one gene specific primer. In the lane, (5) we used the myb74_RP and myb74_LP to confirm homozygosity. **C.** Phenotype of wild-type (Col-0) and *myb74* mutant plants grown for 2 weeks on ½ MS plates under control conditions. **D.** Quantitative RT-PCR analysis of *MYB74* expression in *myb74* relative to *MYB74* expression levels in wild-type Arabidopsis. Corresponding primer sequences can be found in **Supplementary Table S1**.

Figure S2:

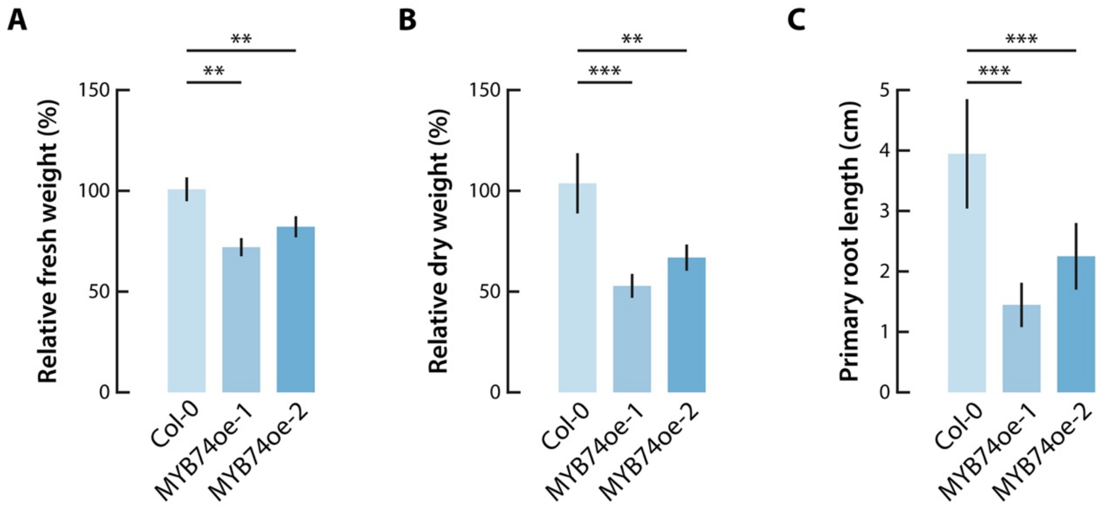

Figure S2. Phenotypic analysis of plants conditionally overexpressing *MYB74*. **A.** Relative fresh weights of the aerial parts of 4 weeks old wild-type control plants (Col-0) and the two MYB74oe lines. The average weight of the Col-0 plants was set as 100%. **B.** To determine the relative dry weight, the same plants were dried in an oven at 60ºC for 48 h, before the weight was again assessed. The experiment was carried out in triplicate with cohorts of n = 50 individual plants per genotype. **C.** Quantitative analysis of primary root length in seven days-old wild-type (Col-0), MYB74oe-1, and MYB74oe-2 plants under control conditions (n = 25). The bar plots represent the means ± SEs of the compared genotypes. Asterisks refer to significant differences between the corresponding wild-type control and the tested MYB74oe lines. Student's t-test: **$p \leq 0.01$, ***$p \leq 0.001$.

Figure S3:

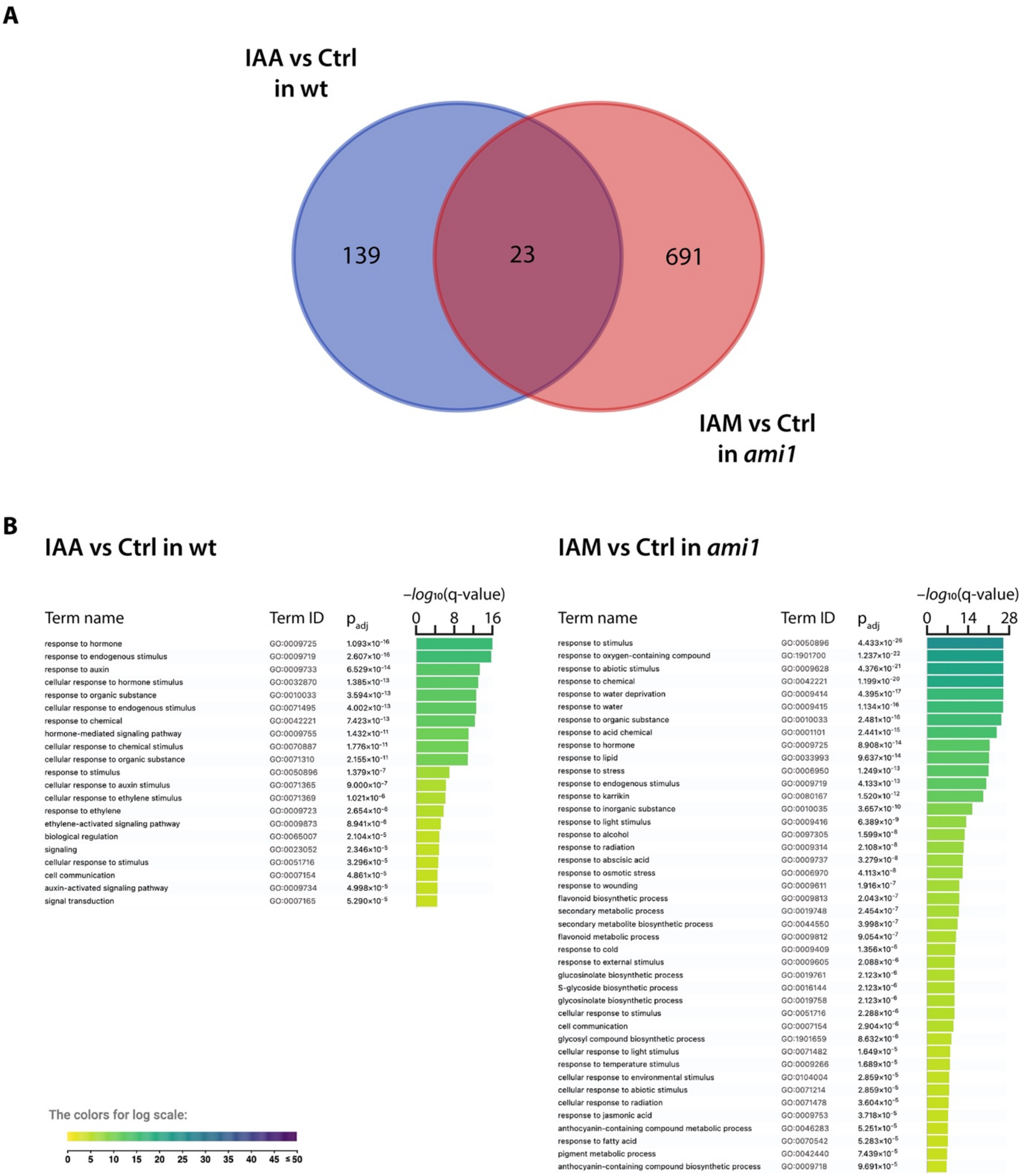

Figure S3. Transcriptomics analysis of differentially expressed genes (DEGs) in IAM versus mock treated *ami1* mutant seedlings (**Supplementary Table S2**) compared to IAA versus mock treated wild-type Arabidopsis seedlings (GSE631) (Okushima et al., 2005). **A.** Venn diagram of differentially regulated genes applying a significance threshold of $q < 0.05$ and a $log_2FC = \pm 1.75$. The two compared datasets share only a minor number of common transcripts. B. Bar plots of GO biological function enrichment analysis of the non-overlapping DEGs in

the tow compared conditions. Color and length of the bars indicate the significance of the identified GO terms. The color code use to differentiate the significance levels is given at the bottom of the figure. The bars only show terms with a significance score -log_{10}(q-value) ≥ 5.

Okushima, Y., Mitina, I., Quach, H.L., and Theologis, A. (2005). AUXIN RESPONSE FACTOR 2 (ARF2): a pleiotropic developmental regulator. *Plant J.* 43(1)**,** 29-46. doi: 10.1111/j.1365-313X.2005.02426.x.

Figure S4:

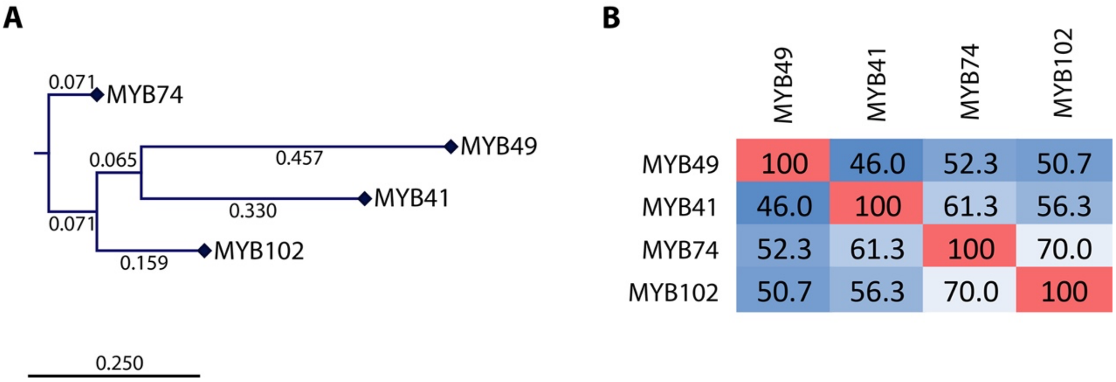

Figure S4. Phylogenetic analysis of the subgroup 11 members of Arabidopsis thaliana R2R3 MYB transcription factor proteins. **A.** Phylogram showing a Neighbor joining tree inferred using the CLC Main Workbench v7.9.2 (Qiagen, Hilden, Germany) with default settings. **B.** Primary amino acid sequence percent identity matrix calculated using the Clustal Omega online tool (https://www.ebi.ac.uk/Tools/msa/clustalo/).
